# Age-Friendly Momentum in Mississippi

**DOI:** 10.1093/geroni/igaf122.352

**Published:** 2025-12-31

**Authors:** Kina White

**Affiliations:** Mississippi State Department of Health, Jackson, Mississippi, United States

## Abstract

The Mississippi State Department of Health (MSDH) is advancing healthy aging through emerging public health policy and practice. While the overall state’s population is declining, between 2010 and 2021, the 65+ population of adults experienced a 29.3% increase. In partnership with Trust for America’s Health (TFAH) and The John A. Hartford Foundation (JAHF), MSDH adopted the 6C’s Framework for Creating an Age-Friendly Public Health Systems (AFPHS) Framework and established a systematic approach to supporting older health and well-being. These efforts resulted in the achievement of exemplar status as a recognized Age-Friendly Public Health System. To achieve AFPHS Recognition, MSDH established a multi-sectoral Advisory Committee and developed a state-wide action plan based on the AFPHS 6C’s Framework using the collective impact process; developed a Healthy Aging Data Report (HADR) and county data profiles with over 120 indicators; partnered with AARP MS and the Mississippi Public Health Association on several endeavors, including the development of a champion program for older adult volunteers to encourage their communities to be age-friendly and promote intergenerational activities. At the intersection of policy and practice, HADRs were hand-delivered to mayors across all 82 counties, translating findings of chronic disease and disability indicators to advance policies that establish age-friendly communities and health systems to improve older adult health outcomes.

